# Antimicrobial Resistance, Virulence Factors and Genetic Diversity of *Escherichia coli* Isolates from Household Water Supply in Dhaka, Bangladesh

**DOI:** 10.1371/journal.pone.0061090

**Published:** 2013-04-03

**Authors:** Prabhat Kumar Talukdar, Mizanur Rahman, Mahdia Rahman, Ashikun Nabi, Zhahirul Islam, M. Mahfuzul Hoque, Hubert P. Endtz, Mohammad Aminul Islam

**Affiliations:** 1 Food Safety Research Group, Centre for Food and Waterborne Diseases, International Centre for Diarrhoeal Disease Research, Bangladesh (icddr,b), Mohakhali, Dhaka, Bangladesh; 2 Department of Microbiology, University of Dhaka, Dhaka, Bangladesh; 3 Department of Medical Microbiology and Infectious Diseases, Erasmus MC, University Medical Center Rotterdam, Rotterdam, The Netherlands; University of Maryland School of Medicine, United States of America

## Abstract

**Background:**

Unsafe water supplies continue to raise public health concerns, especially in urban areas in low resource countries. To understand the extent of public health risk attributed to supply water in Dhaka city, Bangladesh, *Escherichia coli* isolated from tap water samples collected from different locations of the city were characterized for their antibiotic resistance, pathogenic properties and genetic diversity.

**Methodology/Principal Findings:**

A total of 233 *E. coli* isolates obtained from 175 tap water samples were analysed for susceptibility to 16 different antibiotics and for the presence of genes associated with virulence and antibiotic resistance. Nearly 36% (*n* = 84) of the isolates were multi-drug(≥3 classes of antibiotics) resistant (MDR) and 26% (*n* = 22) of these were positive for extended spectrum β-lactamase (ESBL). Of the 22 ESBL-producers, 20 were positive for *bla*_CTX-M-15_, 7 for *bla*_OXA-1-group_ (all had *bla*_OXA-47_) and 2 for *bla*_CMY-2_. Quinolone resistance genes, *qnrS* and *qnrB* were detected in 6 and 2 isolates, respectively. Around 7% (*n* = 16) of the isolates carried virulence gene(s) characteristic of pathogenic *E. coli*; 11 of these contained *lt* and/or *st* and thus belonged to enterotoxigenic *E. coli* and 5 contained *bfp* and *eae* and thus belonged to enteropathogenic *E. coli*. All MDR isolates carried multiple plasmids (2 to 8) of varying sizes ranging from 1.2 to >120 MDa. Ampicillin and ceftriaxone resistance were co-transferred in conjugative plasmids of 70 to 100 MDa in size, while ampicillin, trimethoprim-sulfamethoxazole and tetracycline resistance were co-transferred in conjugative plasmids of 50 to 90 MDa. Pulsed-field gel electrophoresis analysis revealed diverse genetic fingerprints of pathogenic isolates.

**Significance:**

Multi-drug resistant *E. coli* are wide spread in public water supply in Dhaka city, Bangladesh. Transmission of resistant bacteria and plasmids through supply water pose serious threats to public health in urban areas.

## Introduction

Diarrheal diseases account for an estimated 4.1% of the total daily global burden of disease and are responsible for the deaths of 1.8 million people every year, 90% of them are children under the age of 5 [1]. It was estimated that 88% of this burden is attributable to unsafe water supply, sanitation and hygiene, and is mostly concentrated in children in developing countries. *Escherichia coli* is widely used as an indicator organism for the microbiological quality of water is also an important causative agent of diarrhea and other enteric diseases. While most *E. coli* are generally harmless, certain strains of *E. coli* have virulence properties that may account for life threatening infections. Currently, six *E. coli* pathotypes are recognized that can cause diarrhea in humans [2]: enteropathogenic *E. coli* (EPEC), enteroinvasive *E. coli* (EIEC), Shiga toxin-producing *E. coli* (STEC), enteroaggregative *E. coli* (EAEC), enterotoxigenic *E. coli* (ETEC) and diffusely adhering *E. coli* (DAEC). A very few studies have provided adequate information on the occurrence of pathogenic *E. coli* in household waters [3], [4].

Antimicrobial resistance among enteropathogens, including *E. coli* has been reported to be increasing in recent years [5], sometimes leading to point-break situations where no antibiotic treatment options remain [6]. These situations are of serious concern in developing countries where enteropathogens are frequently encountered and cause life-threatening infections, especially among children. The recent emergence and spread of a novel carbapenemase, New Delhi Metallo β-lactamase (NDM)-producing organisms is an example of that situation where available antibiotics are ineffective [7]. This novel enzyme along with other antibiotic resistance factors is carried by mobile genetic elements such as plasmids or transposons [8]. Horizontal gene transfer (HGT) is one of the most common mechanisms by which antibiotic resistance traits are transferred from one organism to another. In *Enterobacteriaceae*, plasmids are the major vectors for HGT. In vivo transfer of resistance traits between *Enterobacteriaceae* in natural ecosystem has been reported [9]. Generic *E. coli* are frequently used as indicator bacteria to monitor the trends in antimicrobial resistance because they are the prevalent commensal enteric bacteria in humans and animals, can be cultured easily and inexpensively [10], and they can acquire and preserve resistance genes from other organisms in the environment and in animal populations [11]. *E. coli* is also considered as a good indicator of the selective pressure imposed by antimicrobial use in food animals [12], [13].

Diarrheal diseases are endemic in Bangladesh. In 2008, an estimated 20,000 children less than 5 years old died of diarrheal diseases in Bangladesh [14]. *E. coli* is one of the leading causes of enteric infection in Bangladesh and ETEC is the predominant pathotype followed by EPEC, EAEC and STEC [15]. Moreover, the dissemination of ESBL and carbapenemases (CARBase) conferring resistance to life-saving β-lactams is of particular concern. Majority of the *E. coli* infections are waterborne as surface water is heavily contaminated with this organism. Poor sanitation and hygiene, overcrowded situation and lack of access to safe drinking water are the precipitating factors. In this study *E. coli* strains isolated from household water supply in Dhaka city were characterized for their antibiotic resistance, pathogenic types, ESBL phenotype, presence of major ESBL genes, and acquisition of transferrable plasmids.

## Materials and Methods

### Sampling site and sample collection

Water samples were collected from the south part of Dhaka city comprising an area of approximately 13 Km^2^ with an approximate population of 300,000. Dhaka is one of the fasted growing mega cities in the world which has an estimated population of 12.5 million living in an area of approximately 350 Km^2^. The areas that we covered under this study were mostly densely populated with low income people and have little water and sanitation facilities. A large urban slum was also included in the sampling area. Water supplied by the municipal authority was the only source of water for the population living in these areas. According to Dhaka Water Supply and Sewerage Authority (Dhaka WASA), around 87% of water supplied by the authority is from ground water abstraction using approximately 605 deep tube wells located in different places of the city and the remaining water comes from surface water treatments.

A total of 175 tap water samples were collected between November 2008 and July 2009 at points of uses in the community. From each point 500 ml of water sample were collected in pre-sterilized Nalgene sampling bottle and samples were transported to the laboratory within 3–4 h maintaining a cool chain.

### Estimation of fecal coliform bacteria and isolation of *E. coli*

Number of fecal coliforms (FC) was estimated in water samples by membrane filtration method according to the procedures described earlier [16]. Briefly, a 100 ml aliquot of water sample was filtered through a 0.2 µm-pore-size membrane filter (Sartorius Stedim, Goettingen, Germany), and the filter was placed on a membrane fecal coliform (MFC) agar (BD, MD, USA) plates. MFC plates were incubated at 44°C for 18–24 h. After incubation, blue colonies which are typical of coliform bacteria were counted and expressed as colony forming units (CFU) per ml of water.

For *E. coli*, a 100 ml aliquot of water sample was filtered according to the procedure as described. The filter was immersed into EE broth (Oxoid ltd, Basingstoke, UK) and incubated at 37°C for 18–24 h. Enrichment broth was cultured on TBX agar medium (Oxoid) and incubated at 37°C for 18–24 h. Typical *E. coli* colonies from TBX plates were picked up and cultured on Eusine Methylene Blue agar (Oxoid) and MacConkey agar medium (Oxoid). From each sample, a maximum of 3 *E. coli* colonies were selected and stored at −70°C for further analysis.

### Antimicrobial susceptibility tests

Susceptibility to antimicrobials was determined by an agar diffusion test using antimicrobial agents impregnated paper discs (Oxoid) as described by the Clinical Laboratory Standards Institute (CLSI) guidelines [17]. The antibiotics used in this study were ampicillin (10 µg), ceftriaxone (30 µg), chloramphenicol (30 µg), ciprofloxacin (5 µg), trimethoprim-sulfamethoxazole (25 µg), gentamicin (10 µg), mecillinam (25 µg), meropenem (10 µg), nalidixic acid (30 µg), tetracycline (30 µg), norfloxacin (10 µg), imipenem (10 µg), kanamycin (30 µg), erythromycin (15 µg), cefotaxime (30 µg), cefixime (5 µg), aztreonam (30 µg), ceftazidime (30 µg), cefoxitin (30 µg) and piperacillin-tazobactam (110 µg). *E. coli* ATCC 25922 and *Staphylococcus aureus* ATCC 25923 were used as negative and positive controls, respectively. CLSI breakpoints were used to interpret the results [17]. Isolates that showed resistance or intermediate susceptibility to cephalosporins were tested for the presence of ESBL by doing double disc synergy test (DDST). The DDST was carried out on Mueller-Hinton agar (Difco Laboratories, Detroit, MI, USA) with discs containing 30 µg of ceftazidime, cefotaxime, or aztreonam, placed at a distance of 15 mm (center to center) from a disc containing amoxicillin-clavulanic acid (20 µg/10 µg) located in the center of the plate [17].

### Detection of antibiotic resistance genes in ESBL-producing organisms

All ESBL-producing isolates were tested for the presence of *bla*_ESBL_ genes (*bla*_TEM_, *bla*_SHV_, *bla*_CTX-M-1-group_, *bla*_CTX-M-15_, *bla*_CTX-M-2-group_, *bla*_CTX-M-8-group_, *bla*_CTX-M-9-group_), carbapenemase genes (*bla*_OXA-1-group_, *bla*_OXA-47_, and *bla*_NDM-1_) and ampC β-lactamase gene *bla*_CMY-2_ by PCR according to procedures described earlier [18]. PCR products of *bla*_CTX-M-15_ primers were sequenced using an ABI PRISM 310 sequencer (Applied Biosystems) in order to confirm the specificity of the gene. In addition, isolates were tested for 16sRNA methyltransferase genes (*rmtA*, *rmtB* and *armA*) and *qnr* genes (*qnrA*, *qnrB* and *qnrS*) according to procedures described earlier [18]. The primer sequences and corresponding annealing temperature used in the PCR reactions are listed in Table 1.

**Table 1:** PCR primers used in the study.

Target gene	Primer	Nucleotide sequence (5′–3′)	Annealing temp (°C)	Product size (bp)
*est*A	ST-F	GCTAAACCAGTA^G^_A_GGTCTTCAAAA	57	147
	ST-R	CCCGGTACA^G^_A_GCAGGATTACAACA		
*elt*B	LT-F	CACACGGAGCTCCTCAGT C	57	508
	LT-R	CCC CCA GCC TAG CTT AGT TT		
*bfp*A	bfpA-F	GGAAGTCAAATTCATGGGGG	57	300
	bfpA-R	GGAATCAGACGCAGACTGGT		
*eae*	eae-F	CCCGAATTCGGCACAAGCATAAGC	57	881
	eae-R	CCCGGATCCGTCTCGCCAGTATTCG		
*aai*C	aaiC-F	ATTGTCCTCAGGCATTTCAC	57	215
	aaiC-R	ACGACACCCCTGATAAACAA		
*aat*	_P_cvd432-F	CTGGCGAAAGACTGTATCAT	57	650
	_P_cvd432-R	CAATGTATAGAAATCCGCTGTT		
*stx*1	stx1F	CACAATCAGGCGTCGCCAGCGCACTTGCT	58	606
	stx1R	TGTTGCAGGGATCAGTGGTACGGGGATGC		
*stx*2	stx2F	CCACATCGGTGTCTGTTATTAACCACACC	58	372
	stx2R	GCAGAACTGCTCTGGATGCATCTCTGGTC		
*iaa*	ial upper	CTGGATGGTATGGTGAGG	57	320
	ial lower	GGAGGCCAACAATTATTTCC		
*ipa*H	Shig-1	TGGAAAAACTCAGTGCCTCT	57	424
	Shig-2	CCAGTCCGTAAATTCATTCT		
*bla*_TEM_	TEM-F	TCGGGGAAATGTGCGCG	57	850
	TEM-R	TGCTTAATCAGTGAGGACCC		
*bla*_SHV_	SHV-F	CACTCAAGGATGTATTGTG	57	861
	SHV-R	TTAGCGTTGCCAGTGCTCG		
*bla*_CTX-M-1 group_	M13-upper	GGTTAAAAAATCACTGCGTC	54	866
	M13-lower	TTGGTGACGATTTTAGCCGC		
*bla*_CTX *-*M-2 group_	M25-upper	ATGATGACTCAGAGCATTCG	56	866
	M25-lower	TGGGTT ACGATTTTCGCCGC		
*bla*_CTX *-*M-8 group_	CTXM8 -F	TCGCGTTAAGCGGATGATGC	58	688
	CTXM8-R	AACCCACGATGTGGGTAGC		
*bla*_CTX-M-9 group_	M9-upper	ATGGTGACAAAGAGAGTGCA	56	870
	M9-lower	CCCTTCGGCGATGATTCTC		
*bla*_CTX-M-15_	CTX-M-15-SF	CACACGTGGAATTTAGGGACT	56	996
	CTX-M-15-SR	GCCGTCTAAGGCGATAAACA		
*bla*_OXA-1 group_	OXA-1F	ACACAATACATATCAACTTCGC	56	814
	OXA-1R	AGTGTGTTTAGAATGGTGATC		
*bla*_OXA-47_	OXA-1A	TCAACTTTCAAGATCGCA	48	609
	OXA-1B	GTGTGTTTAGAATGGTGA		
*bla*_CMY-2_	Forward	GACAGCCTCTTTCTCCACA	50	1143
	Reverse	TGGAACGAAGGCTACGTA		
*bla*_NDM-1_	NDM-F	GGTTTGGCGATCTGGTTTTC	57	465
	NDM-R	CGGAATGGCTCATCACGATC		
*rmt*B	rmtBF	GCTTTCTGCGGGCGATGTAA	55	173
	rmtBR	ATG CAA TGC CGC GCT CGT AT		
*rmt*C	rmtC-F	CGA AGA AGT AAC AGC CAA AG	55	711
	rmtC-R	ATC CCA ACA TCT CTC CCA CT		
*arm*A	armAF	ATT CTG CCT ATC CTA ATT GG	55	315
	armAR	ACC TAT ACT TTA TCG TCG TC		
*qnr*A	QnrAm-F	AGAGGATTTCTCACGCCAGG	56	580
	QnrAm-R	TGCCAGGCACAGATCTTGAC		
*qnr*B	QnrBm-F	GGMATHGAAATTCGCCACTG	56	264
	QnrBm-R	TTTGCYGYYCGCCAGTCGAA		
*qnr*S	QnrSm-F	GCAAGTTCATTGAACAGGGT	56	428
	QnrSm-R	TCTAAACCGTCGAGTTCGGCG		

### PCR for virulence genes

All isolates were examined for the presence of the heat labile (*lt*), heat stable (*st*), attaching and effacing gene (*eae*), bundle forming pilus (*bfp*), antiaggregation protein transporter gene (*aat*) and gene for AggR-activated island (*aaiC*) by multiplex PCR assay. DNA was prepared from overnight grown culture by boiling method. The respective 3 µl template DNA was suspended in 22 µl of reaction mix containing 2.5 µl of 10X PCR buffer with 0.75 µl MgCl_2_, 0.5 µl of 10 mM dNTPs, 0.4 µl each of *lt*, *st*, *bfp*, *aat*, *aaiC* primers, 0.44 µl of *eae* primers, together with 1 unit of *Taq* DNA polymerase (5 U/µl). PCR cycling conditions consisted of initial denaturation at 96°C for 4 min, followed by 34 cycles each of denaturation at 95°C for 20 s, annealing at 57°C for 20 s and extension at 72°C for 1 minute. A separate multiplex PCR for Shiga toxin genes (*stx*_1_ and *stx*_2_) was carried out according to the procedure described earlier [19]. PCR to demonstrate the presence of invasion associated locus (*ial*) and the invasion plasmid antigen H (*ipaH*) was performed according to published procedures [20], [21]. Primer sequences are listed in Table 1.

### Plasmid profile analysis and conjugation experiment

Plasmid DNA was prepared using the rapid alkaline lysis method [22] and analysed by horizontal electrophoresis in 0.7% agarose gels. The molecular weight size of unknown plasmids was estimated by comparing with plasmids that have been used as size standards in the gel electrophoresis. The plasmids Sa (23 MDa), RP4 (34 MDa), R1 (62 MDa), pDK9 (140 MDa) and *E. coli* V517 plasmids (1.4, 1.8, 2.0, 2.6, 3.4, 3.7, 4.8 and 35.8 MDa) were used as standards [23]. Conjugation was carried out by both broth mating and filter mating assays at 30°C using MDR water isolates as donor and *E. coli* MC1061 (Sm^R^, F^−^, non-lactose fermenting) and *E. coli* J53 (Azi^R^, F^−^) as recipients. *E. coli* MC1061 transconjugants were selected on MacConkey agar containing ampicillin (50 mg/L), while the *E. coli* J53 transconjugants were selected on MacConkey agar containing sodium azide (100 mg/L) and cefotaxime (20 mg/L)/cefoxitin (16 mg/L). The duration of conjugation was 18 h. Transconjugant colonies were confirmed by antibiotic susceptibility tests. Plasmid DNA from transconjugants was extracted using alkaline lysis method as described previously [22]. Conjugation frequency per recipient was expressed by dividing the number of transconjugants by the initial number of recipients.

### Genetic fingerprinting

All pathogenic isolates (*n* = 16) were selected for analysis by Pulsed-field gel electrophoresis (PFGE). Genomic DNA was prepared in agarose blocks and digested with the restriction enzyme *Xba*I (New England Biolabs). DNA fragments were separated by pulsed-field gel electrophoresis on a CHEF-MAPPER apparatus (Bio-Rad) according to the PulseNet program developed for *E. coli* [24]. Analysis of the TIFF images was carried out by the BioNumerics software (Applied Maths) using the dice coefficient and unweighted-pair group method using average linkages to generate dendrograms with 1.0% tolerance values.

## Results

### Enumeration of fecal coliform bacteria and isolation of *E. coli*

Around 80% (*n* = 139) of the water samples were positive for fecal coliform (FC) bacteria and 38% (*n* = 67) had a fecal coliform count of >100 CFU/ml of water. *E. coli* was isolated from 63% (*n* = 110) of samples. A total of 233 *E. coli* were isolated from 110 samples that were characterized in this study.

### Antimicrobial susceptibility tests

Of the 233 isolates tested, 57% (*n* = 133) were resistant to ampicillin, followed by 45% (*n* = 105) to tetracycline, 37% (*n* = 87) to nalidixic acid, 36% (*n* = 83) to trimethoprim-sulfamethoxazole, 17% (*n* = 39) to ciprofloxacin, 9% (*n* = 22) to ceftriaxone, 9% (*n* = 20) to mecilinam, 8% (*n* = 18) to chloramphenicol and 1% (*n* = 2) to gentamicin. More than 73% (*n* = 171) of the isolates were resistant to at least one antibiotic and 36% of the isolates (*n* = 84) were resistant to three or more classes of antibiotics thus defined as multi-drug resistant (MDR). Further testing of the 22 ceftriaxone resistant isolates revealed that all were ESBL-producing as confirmed by the double disc synergy test. All the 22 isolates were resistant to cefotaxime and cefixime, 82% to erythromycin, 64% to aztreonam, 55% to ciprofloxacin/norfloxacin, 32% to kanamycin and ceftazidime, 14% to piperacillin-tazobactam and 9% to cefoxitin. None of the isolates were resistant to carbapenem antibiotics, including imipenem and meropenem.

### Detection of antibiotic resistance genes in ESBL-producing organisms

Of the 22 ESBL producing isolates, 20 (90%) were positive for *bla*_CTX-M-1-group_ specific gene and *bla*_CTX-M-15_. Presence of *bla*_CTX-M-15_ gene was confirmed by sequencing the PCR product. One isolate was positive for *bla*_CTX-M-9-group_ specific gene and none of the isolates was positive for *bla*_CTX-M-2-group_, *bla*_CTX-M-8-group_ specific genes. Around 41% (*n* = 9) isolates were positive for *bla*_TEM_ and none were positive for *bla*_SHV_. Among carbapenemase genes, *bla*_OXA-1-group_ and *bla*_OXA-47_ were detected in 32% (*n* = 7) of the isolates. None of the isolates were positive for metallo-β-lactamase gene *bla*_NDM-1_. Plasmidic ampC-type β-lactamases *bla*_CMY-2_ was detected in 9% (*n* = 2) of the isolates. Among quinolone resistance genes, *qnrS* and *qnrB* were detected in 27% (*n* = 6) and 9% (*n* = 2) of the isolates, respectively (Table 2).

**Table 2:** Antibiotic resistance pattern, presence of antibiotic resistance genes and plasmid patterns of ESBL-producing *E. coli* isolated from water samples.

Serial no.	Strain ID	Antibiotic resistance pattern^a^	Presence of ESBL genes	Plasmid pattern (in MDa)
1	4C3	Amp, Cro, Cfm, Ctx	*bla*_CTX-M-15_, *qnr*S	36
2	24C2	Amp, Cip, Cro, Sxt, NA, Te, Cfm, Ctx, Nor, K, E	*bla*_CTX-M-15_, *bla*_OXA-1_, *bla*_OXA-47_	90,3.2,3
3	24C3	Amp, Cip, Cro, Sxt, NA, Te, Mel, Atm, Cfm, Ctx, Nor, E,	*bla*_CTX-M-15_, *bla*_TEM_,	90
4	28C2	Amp, Cip, Cro, Sxt, NA, Te, C, Atm, Caz, Cfm, Ctx, Nor, K, E	*bla*_CTX-M-15_, *bla*_TEM_, *bla*_OXA-1_, *bla*_OXA-47_	105,90,17,2
5	88mf2	Amp, Cro, Mel, Atm, Cfm, Ctx, Tzp	*bla*_CTX-M-15_, *bla*_TEM_	105,90
6	90C1	Amp, Cip, Cro, NA, C, Atm, Cfm, Ctx, Nor, E	*bla*_CTX-M-15_, *bla*_TEM_	62
7	102C1	Amp, Cip, Cro, Sxt, NA, Te, Cfm, Ctx, Nor, E	*bla*_CTX-M-9_	90,8.6,7.4,3.4
8	112C2	Amp, Cip, Cro, Sxt, NA, Te, Fox, Atm, Caz, Cfm, Ctx, Nor, E	*bla*_CTX-M-15_, *bla*_TEM_, *bla*_CMY-2_,	90,35.8,3.1
9	123C4	Amp, Cip, Cro, Sxt, NA, Te, C, Mel, Atm, Caz, Cfm, Ctx, Nor, K, E	*bla*_CTX-M-15_, *bla*_TEM_, *bla*_OXA-1_, *bla*_OXA-47_	140,70
10	134C1	Amp, Cro, Cfm, Ctx, E	*bla*_CTX-M-15_, *qnr*S	140
11	145C2	Amp, Cro, Cfm, Ctx,	*bla*_CTX-M-15_	No Plasmid
12	146C2	Amp, Cro, Cfm, Ctx, E	*bla*_CTX-M-15_, *qnr*S	100
13	156C1	Amp, Cro, Cfm, Ctx, E	*bla*_CTX-M-15_, *qnr*S	140, 62, 27
14	169C1	Amp, Cro, Cfm, Ctx, E	*bla*_CTX-M-15_,	70,2.7
15	169C3	Amp, Cro, Sxt, Te, Atm, Cfm, Ctx, E	*bla*_CTX-M-15_, *bla*_TEM_, *qnr*S	62
16	174TC1	Amp, Cip, Cro, Sxt, NA, Te, C, Cn, Atm, Cfm, Ctx, Nor, K, E	*bla*_CTX-M-15_, *bla*_OXA-1_, *bla*_OXA-47_	105, 2.7,2.1,1.4,1.2
17	174FC1	Amp, Cip, Cro, NA, Te, Atm, Caz, Cfm, Ctx, Nor, K, E, Tzp	*bla*_CTX-M-15_, *bla*_OXA-1_, *bla*_OXA-47_	140,62
18	177TC1	Amp, Cro, Sxt, Te, Atm, Cfm, Ctx, E	*bla*_CTX-M-15_	62
19	185C2	Amp, Cro, Te, Atm, Cfm, Ctx, E	*bla*_CTX-M-15_, *qnr*S	200,100,35.8
20	186C2	Amp, Cip, Cro, Sxt, NA, Te, C, Cn, Atm, Caz, Cfm, Ctx, Nor, K, E, Tzp	*bla*_CTX-M-15_, *bla*_TEM_, *bla*_OXA-1_, *bla*_OXA-47_, *qnr*B	70
21	199C5	Amp, Cip, Cro, NA, Mel, Fox, Atm, Caz, Cfm, Ctx, Nor, E	*bla*_TEM_, *bla*_CMY-2_,	62,23,9
22	200C2	Amp, Cip, Cro, Sxt, NA, Te, Atm, Caz, Cfm, Ctx, Nor, K, E	*bla*_CTXM-15_, *bla*_OXA-1_, *bla*_OXA-47_, *qnr*B	No Plasmid

a Amp, Ampicillin; Atm, Aztreonam; C, Cholramphenicol; Caz, Ceftazidime; Cfm, Cefixime; Cip, Ciprofloxacin; Cn, Gentamycin; Cro, Ceftriaxone; Ctx, Cefotaxime; E, Erythromycin; Fox, Cefoxitin; Imp, Imipenem; K, Kanamycin; Mel, Mecillinam; Mem, Meropenem; NA, Nalidixic acid; Nor, Norfloxacin; Sxt, Sulphamethoxazole-trimethoprim; Te, Tetracycline; and Tzp, Piperacillin-Tazobactam; All S, Sensitive to all antibiotics tested in the study.

### Detection of virulence genes

**Figure 1:**
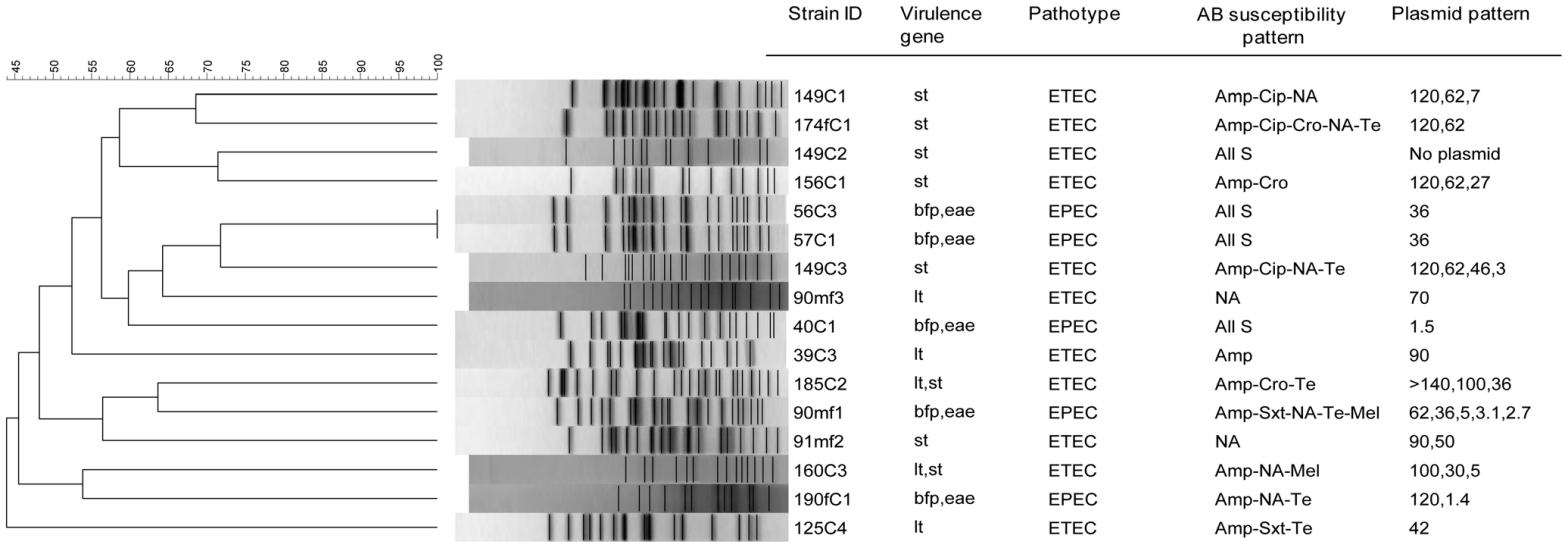
Dendrogram of PFGE fingerprints from pathogenic *E. coli* isolates isolated from water samples. The percentage of genetic homology between banding patterns is indicated. Presence of virulence genes, pathotypes, antibiotic susceptibility pattern and plasmid pattern are plotted next to dendrogram.

Around 7% (*n* = 16) of the isolates were found to be positive for at least one of the 10 pathogenic genes specific to *E. coli* pathotypes. Majority of the isolates (*n* = 11) possessed either *lt* (*n* = 3) or *st* (*n* = 6) or both (*n* = 2), and thus belonged to ETEC. The remaining 5 isolates belonged to EPEC as all of these contained both *bfp* and *eae* genes (Fig. 1).

### Plasmid profile analysis and conjugation experiment

Of the 233 isolates, 186 (80%) carried plasmids of varying sizes generating heterogeneous inter-isolate plasmid profiles. Molecular weights of plasmids ranged from 1.2 MDa to >120 MDa and the number of plasmid ranged from 2 to 8. Conjugation assays with representative isolates (*n* = 15) having different plasmid patterns revealed that plasmids of 50 to 105 MDa were self-transmissible to *E. coli* recipient strain carrying ampicillin resistance. Antibiotic susceptibility tests of transconjugant strains demonstrated that ampicillin resistance conferring plasmids co-transferred trimethoprim-sulfamethoxazole, tetracycline and ceftriaxone resistance. However, resistance to cefoxitin was not transferred via conjugative plasmid. Quinolone resistance determinants were not transferred via conjugative plasmids. Plasmid transfer frequency was nearly the same for all conjugative plasmids expect for a 50 MDa plasmid carrying ampicillin, trimethoprim-sulfamethoxazole and tetracycline resistance, which showed a relatively higher transfer frequency (1.95×10^−2^) than that of the other plasmids (4.4−9.0×10^−4^) (Table 3).

**Table 3:** Results of conjugation assays between antibiotic resistant *E. coli* isolates obtained from water samples and the recipient *E. coli* MC-1061 strain.

Strain no.	Parent strain		Transconjugant		Transfer frequency
	Resistance pattern^a^	Plasmid pattern (MDa)	Resistance pattern^a^	Plasmid pattern (MDa)	
25C3	Amp-Sxt	140,62,45,2.3,2.0	Amp-Sxt	62	9×10^−4^
51C1	Amp-Cip-NA-Sxt-Mel	90,62,4.8,3.7	Amp	62	5.2×10^−4^
88mf2	Amp-Cro-Mel	105, 70	Amp-Cro	70	4.8×10^−4^
133C4	Amp-Sxt-Te	50	Amp-Sxt-Te	50	1.95×10^−2^
174TC1	Amp-Cip-Cro-Sxt-NA-Te-C-Cn	105,2.7,2.1,1.4,1.2	Amp-Cro	105	4.4×10^−4^

a See footnote *a* of Table 2 for definitions of abbreviations.

### Genetic fingerprinting

PFGE analysis of pathogenic *E. coli* isolates revealed diverse banding patterns with similarity indices ranging from 72% to <45%. Two ETEC isolates had identical PFGE patterns and were members of a single clone (Fig. 1).

## Discussion

Household water supply provided by the municipal authority is an important shared resource for millions of people living in Dhaka metropolitan area. *E. coli* is commonly isolated from water sources, including the municipal water supply of Dhaka city [25]. In this study, we found that around 38% of the water samples were contaminated with high counts of fecal coliform bacteria (>100 CFU/ml) and *E. coli* was isolated from 63% of the samples. The presence of *E. coli* in the water sample indicates the presence of microorganisms that might be potentially hazardous for human health and also indicates fecal contamination in water supply system. It becomes a serious threat when these *E. coli* exhibit resistance to multiple antibiotics and pathogenic properties that cause enteric diseases in people who consume this contaminated water. Studies conducted in other countries demonstrated the presence of MDR pathogenic bacteria in water sources including rivers, ponds and lakes [26], [27]. One study from India and another from Canada also reported the presence of antibiotic resistant *E. coli* in drinking water [28], [4].

In the present study, we found that more than 73% of the *E. coli* isolates were resistant to at least one of the 10 antibiotics tested and almost half (49%) of these isolates were multidrug resistant, defined as resistant to three or more classes of antibiotics. Akin to other studies, a higher frequency of resistance against β-lactam, quinolone and floroquinolone antibiotics was observed among the isolates in this study [28], [4]. A significant proportion (9%) of *E. coli* isolates tested in the study was ESBL-producing. This might be due to the residual effect of these antibiotics, which have been used extensively in human population as well as in the food chain creating a selective antibiotic pressure in the environment. A study carried out in 2004 reported that around 43% of *E. coli* isolates obtained from an urban hospital in Dhaka city were ESBL-producing [29].

Among ESBL producers, the majority were positive for clinically significant class A β-lactamases, including *bla*_CTX-M-1-group_, particularly the *bla*_CTX-M-15_. With the beginning of the twenty-first century, *E. coli* strains producing *bla*_CTX-M-15_ have emerged and disseminated worldwide and are now important cause of both nosocomial and community-onset urinary tract and bloodstream infections in humans [30], [31]. The prevalence of CTX-M type β-lactamases in *Enterobacteriaceae* is increasing and in some geographic locations they are now-a-days more prevalent than TEM and SHV types [32]. Both TEM and SHV types have been reported mostly from clinical samples and from some environmental samples like farm animals and estuarine waters [33], [34]. Interestingly, majority of isolates in our study were positive for *bla*_TEM_, while none were positive for *bla*_SHV_. Plasmid mediated quinolone resistance gene, *qnr* has been identified worldwide in different enterobacterial species, including *E. coli* [35], [36], [37]. The prevalence of *qnr* genes, especially *qnrB*, has been reported from clinically important *K. pneumoniae* and other *Enterobacteriaceae* species in Asian countries [38], [39]. Among non-clinical sources, *qnr* has been detected in *E. coli* isolates from livestock, swine and poultry [40], [41]. A *qnrS* gene was identified in a water-borne bacterial species, *Aeromonas*, isolated from the River Seine in Paris [42] and from a Swiss lake [43]. In the present study, we found that two strains were positive for plasmid mediated *qnr* genes of *qnrS* and *qnrB* types. The isolate carrying the *qnrB* co-harbored different classes of β-lactamase genes, including *bla*_CTX-M-15_ and *bla*_OXA-47_ and were resistant to 13 antibiotics, including ciprofloxacin. In contrast, the isolate carrying *qnrS* gene co-harbored *bla*_CTX-M-15_ and *bla*_TEM_ and were resistant to 8 antibiotics, excluding ciprofloxacin. Therefore, the presence of *qnrS* alone may not confer resistance to fluroquinolones as also discussed in previous studies [42].

Many reports have been published on *E. coli* isolates from clinical samples carrying multiple classes of β-lactamases, and metallo β-lactamases [30]. The versatility and fitness of clinically important *E. coli* are proven to acquire most of the variants of β-lactamase genes and the recent acquisition is the New Delhi metallo β-lactamase. A recent study has shown that *E. coli* from environmental sources, including public tap water from New Delhi area, India were positive for multiple classes of β-lactamase, including the NDM-1 [44]. *Enterobacteriaceae* containing NDM-1 gene were also found from the clinical samples in Bangladesh [18]. However, none of the isolates in the present study was positive for NDM-1 gene. Isolates in this study were collected during 2008–2009, the period preceding the emergence of NDM-1. Nevertheless, it is not unlikely that the recent isolates will carry this gene and hence a continued surveillance is warranted. Pathogenic *E. coli* contributes significantly to the burden of infectious diseases in parts of the world where enteric diseases are endemic. Although water is considered as an important route of transmission of pathogenic *E. coli*, only a few published reports are available that describe its transmission via household water supply. In this study, we found that a significant percentage (7%) of *E. coli* isolates from supply water sources belonged to the pathogenic types, including EPEC and ETEC. In Bangladesh, diarrheal diseases are a major health problem, and pathogenic *E. coli* are the second leading causes of diarrhea next to rota virus. ETEC accounts for about 20% of all diarrheal cases in children under 2 years of age [15]. It has been shown previously that ETEC is present in drinking water and environmental water in Dhaka and viable after long-term water incubation which suggests that water might be an important route of transmission [45], [46]. In a recent study it has been shown that ETEC form biofilms in household drinking water which can be found during all months of the year and an increase during summer and rainy season [47]. At present, there is no vaccine available for *E. coli* diarrhea and the treatment modalities, including antibiotic therapy are not very efficient due to the emergence of MDR organisms. It is likely that multiple exposure pathways are involved in transmitting the MDR pathogenic *E. coli* to humans but household water supply play a significant role as it is highly contaminated and people get exposed to contaminated water very easily.

Plasmid profile analysis revealed that the majority (80%) of isolates contained multiple plasmids and there was a little similarity of patterns among the isolates indicating their clonal diversity. Around 14% of the isolates (*n* = 32) contained a large plasmid of >120 MDa. It is established that plasmids of this size carry invasive properties for certain enteropathogens, including *Shigella* spp., and Enteroinvasive *E. coli* (EIEC) [48]. In general, all invasive *Shigella* spp. and EIEC strains are positive for *ipaH* and *ial* genes, which are considered as surrogate markers for the test of invasiveness. None of the large plasmid-containing isolates in the study was positive for *ipaH* and *ial* genes. This accentuates the need for further studies to understand the role of large plasmids in *E. coli* isolates. Analysis of plasmid profile revealed that a large number of isolates (*n* = 122) contained plasmids in the range of 50 to 100 MDa (middle-ranged). It has been demonstrated previously that plasmids of these sizes in *Enterobacteriaceae*, particularly in *Shigella* spp. and *E. coli* are generally self-transmissible and carry the antimicrobial resistance factors [49], [50]. In this study, we also found correlation between the presence of middle ranged plasmids and multi-drug resistance phenotypes among the isolates. We identified self-transmissible plasmids that carry ampicillin resistance in different strains ranging in weight size from 50–105 MDa. Although we selected the conjugative plasmids based on ampicillin resistance, other antibiotic resistance particularly trimethoprim-sulfamethoxazole, tetracycline and ceftriaxone were co-transferred by these plasmids with a different transfer frequency (Table 3). The resistance to cefoxitin in two isolates (112C2 and 199C5) was not transferred by conjugation to the recipient *E. coli* J53, although both isolates were positive for *bla*_CMY-2_, a plasmid-mediated AmpC beta-lactamase. No transfer of ciprofloxacin or nalidixic acid was observed indicating that quionolone groups are not transferrable by conjugative plasmid. Transfer of resistance plasmids by conjugation was not successful for a number of isolates. The plasmid carrying the resistance gene in these isolates may be in non-conjugative plasmids or chromosomally encoded. Further studies are needed to determine the location of resistance genes in these isolates.

A high degree of polymorphism was observed in PFGE patterns of the isolates. A total of 15 distinct profiles were obtained among 16 pathogenic isolates indicating their genetic diversity. Only two ETEC isolates had identical PFGE patterns. Interestingly, both isolates had identical plasmid and antibiotic susceptibility patterns (Fig. 1). Tracing back to the source, we found that these organisms were isolated from water samples obtained from two different points-of-use within the same area where water is supplied from the same point-of-source through a single pipeline. This result indicates the clonal transmission of pathogenic organisms in the community through supply water system.

One of the limitations of the study is that we could not collect water samples from the entire areas of the city. Therefore, the level of contamination of water might not representative of other areas of the city. However, most Dhaka residents rely on the municipal water, which is mainly abstracted from underground sources and circulated to households following the same system. As such, the risk of exposure to MDR pathogenic organisms via water supply system in Dhaka residents would not be significantly different from one area to the other.

The household water supply is normally consumed by people without any pre-treatment, although boiling of water before consumption is often advised. Hence, the presence of multi-drug resistant ESBL-producing pathogenic *E. coli* in household water supply in Dhaka has important implications for health of the urban population. The MDR *E. coli* may represent an important reservoir of genetic determinants of antimicrobial resistance that can easily be transferred to other microorganisms in the environment through HGT, including the potential human pathogens. Effective prevention strategies are needed to limit the widespread circulation of these bacteria in the community and to contain the threat of emerging drug resistance among various enteric bacterial pathogens.
